# Clinical Detoxification: Elimination of Persistent Toxicants from the Human Body

**DOI:** 10.1155/2013/238347

**Published:** 2013-06-06

**Authors:** Stephen J. Genuis, Margaret E. Sears, Gerry Schwalfenberg, Janette Hope, Robin Bernhoft

**Affiliations:** ^1^Faculty of Medicine, University of Alberta, 2935-66 Street, Edmonton, AB, Canada T6K 4C1; ^2^Children's Hospital of Eastern Ontario Research Institute, Ottawa, ON, Canada K1H 8L1; ^3^American Academy of Environmental Medicine, Wichita, KS 67206, USA

Underlying individuals' unique, invaluable, and enigmatic metaphysical qualities, the human organism is, in a physical sense, essentially a self-regulating biochemical machine. At any moment, our thoughts and feelings, our actions, metabolism and physical well-being all stem from the sum of dynamic, intricate biochemistry working within a distinctive genetic context; innumerable biochemical reactions are taking place to prepare the enzymes, hormones, neurotransmitters and all that we need to undertake the tasks required for daily life. We are truly wonderfully crafted. Like any functional system, however, in order to thrive we must receive the raw materials that we need to carry out our biological processes and we must stay away from influences that are harmful and which impair our machine from functioning normally. The widespread introduction of assorted toxic chemical agents into our intricate biochemical workings has the potential to disrupt sophisticated biochemical processes, becoming a widespread source of harm. Early life exposures can have life-long consequences, even at levels commonly experienced and thought to be safe [1, 2]. 

Attention to toxic chemical exposures and environmental health sciences has been expanding at an impressive rate. Extensive research by independent scientists as well as governments has prompted numerous toxicology, medical, public health and other scientific journals to report on the impacts of environmental determinants on human health. With the recognition that recent and emerging changes in the external environment have the potential to influence genetic function, hormonal biology, the gastrointestinal microbiome, and mitochondrial processes, as well as other important physiological parameters, the significance of environmental medicine on individual and population health is rapidly becoming a major area of study for scientists and public health officials. 

A concern that has become increasingly manifest is that persistent toxicants are retained within the human body long after the primary exposure [3]. Many toxic compounds have long half-lives; they biomagnify up the food chain, and some are increasingly found in the air we breathe, water we drink, food we consume, and assorted personal care products we apply to our skin. Moreover, many persistent pollutants accumulate in developing children through vertical transfer from mother to child in utero and via breast milk [4]. As a consequence, many individuals now carry heavy body burdens of persistent toxicants, which often increase with advancing age as a result of ubiquitous exposures. Furthermore, despite some nations' regulations to restrict the ongoing use of some toxicants, historical contamination of persistent pollutants and regional release in other jurisdictions lacking restrictions have resulted in ongoing exposures and bioaccumulation throughout much of the world. 

While the chemical revolution was birthed and grew prolifically over the last 5 to 6 decades, it appears that we will be spending much of the next few decades trying to deal with the fallout of this revolution. Future generations may look back with astonishment and wonder how our culture thought it could stand by and tolerate the poisoning of its people and somehow not anticipate the ravages of widespread disordered biochemistry and ill health. With the mounting severity of the toxicant bioaccumulation problem, however, organizations such as the Pediatric Academic Societies have begun to speak out announcing that “low level exposure to environmental toxicity may be impacting the functioning of the current generation” [5]. Furthermore, with the recognition of the potential damage to children, the World Health Organization recently expressed the urgent need to build “Children's Environmental Health Capacity among Health Care Professionals” [6]. 

Despite recent recognition that accrual of toxicants is a major determinant in many chronic health problems, however, little attention in the mainstream medical literature has been devoted to mechanisms to address and resolve the problem of endogenous chemical accrual. Diminishing the influence of persistent harms has the potential to allow the biochemical machinery to be restored. Intervention to reduce the body burden of persistent toxicants—the field of clinical detoxification—constitutes a fundamental and urgently required approach to reducing toxicant-related health issues. It is rewarding indeed to witness remarkable recoveries from chronic illness that are made possible by removing the toxic etiological sources of harm that are disrupting human molecular biochemistry at a microscopic level and thus inducing clinical illness at a macroscopic level [7–10]. 

The main focus of this special issue is the translation of emerging scientific knowledge in clinical detoxification, in order to provide practical and useful information for clinical medicine as well as public health policy. The disciplines of environmental sciences, toxicology, epidemiology, clinical practice, and public policy mesh in this important field of science. This special issue was envisioned as a starting place for researchers and clinicians to summarize the most recent developments and ideas in the field of clinical detoxification, with a special emphasis given to practical methods to diminish the total load or body burden of toxicants within individuals. 

We sent out a call for papers and, as expected in this nascent field, the response was not overwhelming. The reality is that we are in the early stages of knowledge translation in environmental health sciences. Thus far, there is a dearth of scientists and clinicians who are systematically researching interventions to eliminate persistent toxicants, and many clinicians in mainstream medicine have not yet been apprised of the issue of toxicant bioaccumulation.

Just the same, we received over a dozen submissions, of which five papers within the field of detoxification were chosen for publication. This represents a noble start, exploring a variety of topics. With the common and widespread problem of mold contamination in water damaged buildings, we are pleased to present a comprehensive review paper on the management of mold and mycotoxin exposure. As suspicion and confusion abound among some physicians regarding chelation, we present an insightful review of the broad field of chelation for detoxification of toxic elements. While various papers in the literature on toxic elements have explored the issues of mercury and lead contamination, we are grateful to share a paper on the rising problem of cadmium toxicity.

One of the predominant determinants of persistence of toxic compounds within the body is their level of lipophilicity—or their affinity for fat tissues. Many lipophilic compounds have prolonged half-lives in the body with ongoing potential for detriment. We are pleased to include a paper in this edition on novel interventions designed to facilitate the rapid elimination of some lipophilic compounds through the gastrointestinal tract. Finally, we present a paper which explores the effect of induced sweating on the release of phthalate plasticizers—one of the most common chemical exposures of contemporary society. Thus a variety of problems and potential management strategies are examined and discussed in this special edition.

With the ongoing chemical erosion of human health [11], we anticipate that there will be continued and escalating attention to the area of environmental health sciences in the medical community, as well as among the general population throughout the world. As the impact of toxic chemicals can include health afflictions involving many varied specialties, we also anticipate a need for clinicians from the spectrum of medical disciplines to become aware of this problem and to acquire the knowledge and skills necessary to intervene to diminish toxicant body burdens. As more clinical scientists become apprised of the reality of toxicant bioaccumulation and impacts on health, it is likely that elimination of toxicants or “clinical detoxification” will gain status as a foremost aspect of mainstream medical practice. We hope that this special issue will be a springboard for more research and attention to this field. Thank you for perusing and for considering the information we present in this special issue.

*Stephen J. Genuis* *Stephen J. Genuis* *Margaret E. Sears* *Margaret E. Sears* *Gerry Schwalfenberg* *Gerry Schwalfenberg* *Janette Hope* *Janette Hope* *Robin Bernhoft* *Robin Bernhoft*
